# Default Mode Network, Disorganization, and Treatment-Resistant Schizophrenia

**DOI:** 10.1093/schbul/sbaf018

**Published:** 2025-03-03

**Authors:** Huan Huang, Xuan Qin, Rui Xu, Ying Xiong, Keke Hao, Cheng Chen, Qirong Wan, Hao Liu, Wei Yuan, Yunlong Peng, Yuan Zhou, Huiling Wang, Lena Palaniyappan

**Affiliations:** Department of Psychiatry, Renmin Hospital of Wuhan University, Wuhan 430060, China; Department of Psychiatry, Douglas Mental Health University Institute, McGill University, Montreal, Quebec H4H 1R3, Canada; Department of Psychiatry, Renmin Hospital of Wuhan University, Wuhan 430060, China; Department of Psychiatry, Renmin Hospital of Wuhan University, Wuhan 430060, China; Department of Psychiatry, Renmin Hospital of Wuhan University, Wuhan 430060, China; Department of Neurobiology and Department of Psychiatry of the Second Affiliated Hospital, Zhejiang University School of Medicine, Hangzhou 310009, China; Department of Psychiatry, Renmin Hospital of Wuhan University, Wuhan 430060, China; Department of Psychiatry, Renmin Hospital of Wuhan University, Wuhan 430060, China; Department of Psychiatry, Renmin Hospital of Wuhan University, Wuhan 430060, China; Department of Psychiatry, Yidu People’s Hospital, Yidu 443300, China; Department of Psychiatry, Yidu People’s Hospital, Yidu 443300, China; Institute of Psychology, Chinese Academy of Sciences, Beijing 100101, China; Department of Psychiatry, Renmin Hospital of Wuhan University, Wuhan 430060, China; Hubei Provincial Key Laboratory of Developmentally Originated Disease, Wuhan 430071, China; Department of Psychiatry, Douglas Mental Health University Institute, McGill University, Montreal, Quebec H4H 1R3, Canada; Department of Psychiatry, Schulich School of Medicine and Dentistry, Western University, London, Ontario N6C 0A7, Canada; Robarts Research Institute, Schulich School of Medicine and Dentistry, Western University, London, Ontario N6A 3K7, Canada

**Keywords:** predictability, complexity, triple network, clozapine, formal thought disorder, psychosis

## Abstract

**Background and Hypothesis:**

Disorganized thinking is a prominent feature of schizophrenia that becomes persistent in the presence of treatment resistance. Disruption of the default mode network (DMN), which regulates self-referential thinking, is now a well-established feature of schizophrenia. However, we do not know if DMN disruption affects disorganization and contributes to treatment-resistant schizophrenia (TRS).

**Study Design:**

This study investigated the DMN in 48 TRS, 76 non-TRS, and 64 healthy controls (HC) using a spatiotemporal approach with resting-state functional magnetic resonance imaging. We recovered DMN as an integrated network using multivariate group independent component analysis and estimated its loading coefficient (reflecting spatial prominence) and Shannon Entropy (reflecting temporal variability). Additionally, voxel-level analyses were conducted to examine network homogeneity and entropy within the DMN. We explored the relationship between DMN measures and disorganization using regression analysis.

**Results:**

TRS had higher spatial loading on population-level DMN pattern, but lower entropy compared to HC. Non-TRS patients showed intermediate DMN alterations, not significantly differing from either TRS or HC. No voxel-level differences were noted between TRS and non-TRS, emphasizing the continuum between the two groups. DMN's loading coefficient was higher in patients with more severe disorganization.

**Conclusions:**

TRS may represent the most severe end of a spectrum of spatiotemporal DMN dysfunction in schizophrenia. While excessive spatial contribution of the DMN (high loading coefficient) is specifically associated with disorganization, both excessive spatial contribution and exaggerated temporal stability of DMN are features of schizophrenia that become more pronounced with refractoriness to first-line treatments.

## Introduction

Schizophrenia significantly impairs daily functioning and leads to substantial disability worldwide. Despite introducing novel antipsychotic treatments, approximately one-third of individuals become treatment-resistant (TRS), characterized by an inadequate response to at least 2 different first-line antipsychotic drugs.^1–3^ Clozapine, a second-line antipsychotic, is the only evidence-based pharmacological option for TRS. The initiation of clozapine is often delayed, worsening the treatment refractoriness,^4^ but we are not able to use it pre-emptively due to the lack of reliably detectable mechanistic features of treatment resistance.

Over the past 3 decades, neuroimaging techniques have offered valuable insights into the neural pathophysiology of schizophrenia.^5–9^ In particular, the reliability of measuring large-scale resting-state networks (RSNs) such as the default mode network (DMN)^10–12^ has raised the potential for detecting latent markers of treatment resistance.^13–17^ The DMN is essential for internally directed cognitive processes, especially in organizing self-referential thoughts. Disruptions of the DMN in schizophrenia^18^ manifest as regional anatomical/functional abnormalities,^19^ aberrant activity/deactivation during a wide range of tasks,^20^ altered pattern of structural and functional connectivity,^21,22^ as well as changes in topological properties of the connectome.^23,24^ Antipsychotics have a notable effect on the DMN, with disruptions being more pronounced in individuals not responding to these interventions.^8,25,26^

DMN abnormalities have also been associated with core symptoms of schizophrenia,^27^ including the disorganization^28,29^—a symptom cluster characterized by incoherent thinking and speech. Disorganization is one of the most genetically influenced symptoms of schizophrenia,^30–32^ with its onset often preceding the first psychotic episode^33,34^ and persistence extending over several years, notably affecting daily functioning^35,36^ (for a review, see Palaniyappan^37^). Disorganization predicts the onset of treatment resistance.^38,39^ In an extensive 10-year follow-up of 102 first-episode patients, baseline severity of disorganization was the only symptom domain that predicted eventual treatment resistance.^40^ In a 15-year follow-up, McGlashan reported a “drift toward disorganization” as a notable feature of persistent symptoms in schizophrenia.^41^ Thus, a putative pathophysiological substrate of treatment refractoriness, such as DMN dysfunction, can be expected to influence the severity of disorganization.

Currently, 2 main methods are employed to derive RSNs such as the DMN from functional MRI (fMRI) data: Hypothesis-driven methods, such as seed-based approaches, and data-driven methods, such as group-independent component analysis (gICA).^42^ The former relies on predefined regions of interest or brain parcellations (ie, atlas),^43,44^ providing clear interpretations but constrained by prior knowledge about the brain’s intrinsic functional architecture. In contrast, the latter does not require such assumptions, allowing for a more nuanced and unbiased exploration of brain networks by identifying independent components (ICs) from the data itself.^45,46^ Due to its multivariate approach and ability to uncover intricate patterns, gICA has been increasingly adopted as a trustworthy method for restoring common brain networks like the DMN, especially in complex disorders like schizophrenia.^21,22,47–49^ Moreover, gICA quantifies intersubject variability through loading coefficients (ie, the degree to which an individual’s fMRI data contributes to a specific network pattern), offering valuable insights into how consistently DMN is represented across different subjects. This is crucial for addressing the heterogeneity in treatment response among TRS patients and identifying DMN covariance patterns linked to treatment resistance. Complementing the spatial perceptive, the temporal complexity of DMN signals can provide critical information regarding its functional integrity.^50^ The importance of such a spatiotemporal examination to study schizophrenia has been argued cogently by Northoff and colleagues.^51–53^ Specifically, brain entropy captures the stability (predictability) and variability of the fMRI signal over time.^54^ This measure, rooted in Shannon’s information theory, offers a nonlinear perspective on the dynamic behavior of neural activity, which when altered in the DMN, could reflect anomalous DMN-centered cognition.

**Figure 1.**
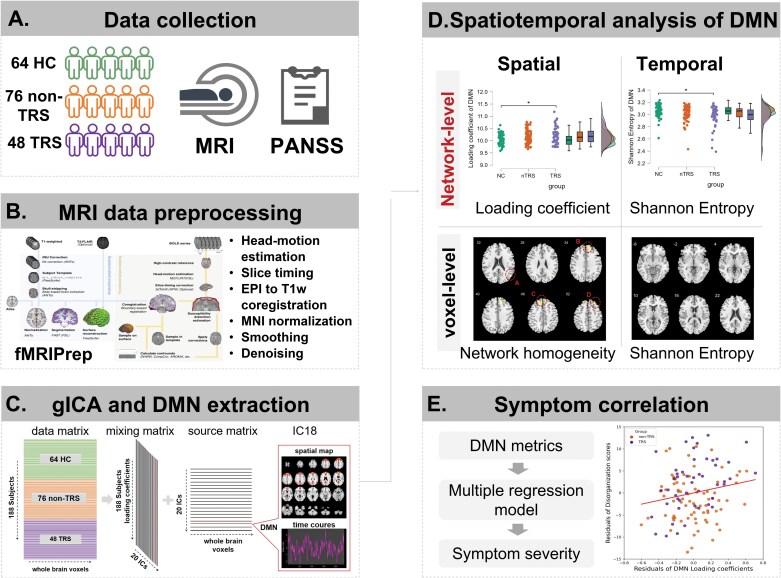
Scheme of Analysis. (A) MRI data and the Positive and Negative Syndrome Scale (PANSS) scores of 64 healthy controls (HC), 76 non-treatment-resistant schizophrenia (non-TRS) patients, and 48 treatment-resistant schizophrenia (TRS) patients were collected. (B) Functional MRI data were preprocessed using fMRIPrep pipeline. (C) Group-independent component analysis (gICA) was performed on the entire dataset to extract the default mode network (DMN). (D) Spatiotemporal analysis of the DMN was conducted including network-level loading coefficients and Shannon entropy, as well as voxel-level network homogeneity and Shannon entropy. (E) Correlations between DMN metrics and symptom severity were explored using multiple regression models.

In this study, we employ a 2-tiered spatiotemporal analysis to investigate DMN abnormalities in a relatively large single-site cohort of individuals with TRS, non-TRS, and healthy controls (HC), and Figure 1 illustrates the overall workflow of the study. We use multivariate gICA to identify and quantify DMN patterns across all individuals, focusing on network-level loading coefficients and Shannon entropy to capture both spatial and temporal aspects of DMN as a whole. Additionally, we conducted voxel-wise analyses to examine network homogeneity and Shannon entropy within DMN, allowing for a more granular voxel-level understanding of the network’s characteristics. Regression analyses were performed to explore the relationship between DMN disruptions and disorganization symptoms, as measured by the Positive and Negative Syndrome Scale (PANSS).^55^ This comprehensive approach aimed to determine whether TRS represents a distinct neurobiological subtype (ie, only seen in TRS but not in non-TRS) or a more severe expression of a continuous DMN dysfunction that varies with the burden of disorganization.

## Methods

### Participants

We recruited 145 right-handed schizophrenia patients from the outpatient and inpatient psychiatry departments at Renmin Hospital of Wuhan University, between August 2020 and December 2022. Patients were independently diagnosed with schizophrenia or schizoaffective disorder based on the DSM-5 criteria by 2 trained psychiatrists, using the Structured Clinical Interview for DSM-5 (SCID). Clinical symptoms of all patients were evaluated through a semi-structured interview using the PANSS by trained psychiatrists. All patients were experiencing an acute episode, defined by a current severity of illness score of 4 or more (moderate) on at least 2 positive symptoms of PANSS, focusing on acute-phase characteristics and symptom severity rather than chronic residual states.

The cohort included 59 TRS and 86 non-TRS patients. Treatment resistance was defined as minimal or no symptomatic response to at least 2 different non-clozapine antipsychotics administered at adequate doses (at least 400 mg/day chlorpromazine equivalent, based on Leucht et al.^56^ and in line with prior and ongoing studies^57,58^) and for adequate duration (minimum of 6 weeks). The determination of treatment resistance was based on comprehensive medical records and detailed clinical interviews conducted by psychiatrists, who assessed the patients’ current and past treatment histories and ensured that intolerance or self-discontinuation was not classified as treatment failure.

Non-TRS patients responded to non-clozapine antipsychotics, with a response defined by a PANSS score of 3 or less (mild) on all positive symptoms and a CGI-S score of 3 or less following acute-phase treatment, sustained during a follow-up period of 6–12 weeks. Moreover, they experienced no symptomatic relapse during the clinical follow-up period, indicating sustained antipsychotic efficacy.

Information on antipsychotic medication was collected for all participants on the day of the MRI scan. Among the non-TRS patients, 34 were in their first episode, of whom 2 were unmedicated at the time of scanning; the remaining non-TRS patients were undergoing antipsychotic treatment. All patients in the TRS group were on 1 or more antipsychotic medications, with 21 having already started clozapine when MRI was obtained. Daily dosages were converted to chlorpromazine (CPZ) equivalents (mg/day) using an established conversion method.^56^ We characterized disorganization symptoms using a composite PANSS factor from the 5-factor structure based on Shafer and Dazzi meta-analysis.^55^

Sixty-six right-handed healthy controls (HC group) were recruited from the local community through advertisements and word-of-mouth. Healthy controls were excluded if they had a history of psychiatric disorders in first-degree relatives. We screened all controls for a lifetime absence of psychiatric disorders using Mini International Neuropsychiatric Interview.

Exclusion criteria for all groups included current or past neurological disorders, substance abuse or dependence, mental retardation, severe and unstable somatic diseases, history of neurosurgery, pregnancy, or lactation, and any MRI contraindications.

Due to various reasons, 23 participants were excluded from the analysis (see Supplementary Materials for details). In total, 188 subjects (64 controls, 76 individuals with non-TRS, and 48 individuals with TRS) were included in the final analysis. The research protocol was approved by the ethics committee of Renmin Hospital of Wuhan University. All participants or their legal guardians provided written, informed consent after receiving a detailed verbal description and explanation of the study.

### MRI Data Acquisition

All MRI data were acquired at the Renmin Hospital of Wuhan University using a 3.0 Tesla GE DISCOVERY MR750w scanner, equipped with a standard 8-channel phase array head coil. Resting-state fMRI data were acquired using a gradient-echo echo-planar imaging (EPI) sequence with the following acquisition parameters: repetition time (TR) = 2000 ms, echo time (TE) = 30 ms, flip angle = 90°, slice thickness = 4 mm with no gap, field of view (FOV) = 220 mm × 220 mm, matrix = 64 × 64, and voxel size = 3.4375 mm × 3.4375 mm × 4 mm. A total of 36 axial slices were acquired, with each scan lasting 8 minutes, resulting in 240 volumes. High-resolution T1-weighted structural images were acquired using a magnetization-prepared rapid acquisition gradient-echo sequence with the following parameters: TR = 8.5 ms; TE = 3.2 ms, flip angle = 12°, FOV = 256 mm × 256 mm; matrix = 256 × 256, and voxel size = 1 mm × 1 mm × 1 mm, covering 176 sagittal slices.

### Imaging Data Preprocessing

All MRI DICOM data were organized into BIDS format using heudiconv v1.1.3 (https://github.com/nipy/heudiconv), with the first 5 time points discarded to account for magnetic field inhomogeneity. The data were subsequently preprocessed using *fMRIPrep v24.0.1* (https://fmriprep.org/en/stable/; RRID:SCR_016216),^59^ which is based on *Nipype 1.8.6* (RRID:SCR_002502).^60^ The preprocessing pipeline included head-motion estimation, slice timing correction, EPI to T1w coregistration, normalization to MNI space. Details can be found in the Supplementary Materials. Mean framewise displacement (FD) was also calculated for each subject using the formulations following Jenkinson,^61^ and the subjects with mean FD greater than 0.2 were removed.

### Spatial Group-Independent Component Analysis and DMN Identification

Spatial gICA was carried out using the GIFT v4.0.5.2 toolbox (http://icatb.sourceforge.net), running on MATLAB_R2023a (https://www.mathworks.com/). Prior to the gICA, spatial smoothing with a 6-mm full-width at half-maximum (FWHM) Gaussian kernel was applied to preprocessed fMRI data. We used the Infomax algorithm to estimate independent sources, which applies multivariate decomposition to separate the fMRI BOLD signal, represented as a large data matrix with time points as rows and voxels as columns, into a set of group-level independent components (ICs) by first concatenating data from all subjects along the time dimension. The minimum description length criterion was used to estimate the number of ICs, resulting in 20. The ICA process was repeated 20 times and the results were clustered by the ICASSO algorithm with both RandInit and Bootstrapping (http://research.ics.aalto.fi/ica/icasso/). We confirmed the consistency and reliability of the resulting ICs as all stability index Iq of >0.9. The ICs representing DMN were identified using a spatial correlation approach, which showed the best match to the predefined DMN templates in GIFT (*r* = 0.5302). The gICA workflow has been displayed in Figure S1. After identifying the group-level DMN, we back-reconstructed the DMN for each subject and calculated the loading coefficients. The DMN loading coefficients quantify the alignment between the group-level DMN pattern and each subject’s specific fMRI data, reflecting how strongly the group-derived DMN pattern is represented in each subject.^62^ Each IC has 2 parts: Spatial map, which represents brain regions that show synchronized activity (ie, an independent functional network), and time series, which illustrate how the activity with the spatial map changes over time.^63–65^

### DMN Network-Level Metrics

We used loading coefficients per subject to describe the degree to which the DMN contributes to an individual’s spatial connectivity map and Shannon entropy to capture DMN temporal complexity/uncertainty over time in each subject. During the back-reconstruction process, the loading coefficients for each subject were calculated by linearly regressing the participant’s fMRI data onto the group-level DMN spatial maps, with the resulting regression coefficients representing the strength of each participant’s contribution to the corresponding group-level DMN pattern. The time courses of the DMN for each subject were detrended and despiked, followed by filtering with a high cutoff of 0.15 Hz. Additionally, we regressed the 24 head-motion parameters, as well as white matter (WM) and cerebrospinal fluid (CSF) signals. Shannon entropy was then computed by assessing the probability distribution of the time series, providing a quantitative measure of the unpredictability and complexity of the DMN’s temporal dynamics.

### DMN Voxel-Level Metrics

We used voxel-wise Network Homogeneity (NH) and Shannon entropy to further analyze the voxel-level properties of the DMN. The DMN masks were generated as in previous studies, see Supplementary Material for a detailed description and Figure S2 for spatial display. Before calculating voxel-level metrics, the fMRI data, which have been minimally preprocessed using *fMRIPrep*, underwent additional processing (https://brainhack-princeton.github.io/handbook/content_pages/05-07-funcConn.html#confound-regression). The steps included detrending, regression of 24 head-motion parameters, WM and CSF signals, along with filtering (0.01–0.1 Hz). Network Homogeneity is a voxel-wise measure used to assess functional connectivity within a specific network, in this case, the DMN, providing an unbiased and objective analysis of the DMN’s internal connectivity. Network Homogeneity is defined as the mean linear Pearson correlation of a given voxel’s time series with those of other voxels in the network. The voxel-wise Shannon entropy was calculated similarly to the network-level analysis, but we computed it for the time series of each individual voxel within the DMN mask. Then both correlation coefficients and entropy values of all voxels were converted into *z*-values to generate NH and entropy *z*-maps of each subject, smoothed by a 6-mm FWHM Gaussian kernel.

### Statistical Analyses

Chi-squared tests, 2-sample *t*-tests, and Analysis of Variance (ANOVA) were used to compare categorical and continuous variables for demographic and clinical data, and the significance threshold was set at *P* < .05. Group comparisons were performed using Analysis of Covariance (ANCOVA), with age, sex, years of education, and mean FD as covariates. For network-level metrics, the significance threshold for ANCOVA was set at *P* < .05, while for voxel-level metrics, Threshold-Free Cluster Enhancement with *P*-FWE < .05 and a minimum cluster size of 10 voxels was applied. Tukey’s LSD test was conducted to further investigate the significant group effects between any pair of the 3 groups. To explore the correlation of disorganization and other clinical variables with DMN metrics, multiple regression analyses were performed with age, sex, years of education, and mean FD as covariates in the TRS and non-TRS groups separately, as well as in the combined full patient group.

### Exploratory Analysis

We conducted an exploratory analysis by selecting the other RSNs simultaneously extracted by ICA, all the above network-level analyses also have been conducted on these RSNs.

## Results

### Demographic and Clinical Information

The demographic and clinical characteristics of 188 participants are summarized in Table 1. There were no significant differences in sex distribution among the 3 groups (χ² = 4.102, *P* = .129.), but age (*F* = 9.604, *P* < .001) and years of education (*F* = 21.619, *P* < .001) differed significantly. The TRS group was older than the non-TRS group, which was matched with the HCs. HC group had significantly more years of education compared to the 2 patient groups. The PANSS scores indicated that the TRS group had higher total scores (*t* = −2.165, *P* = .032), compared to the non-TRS group, as expected. There were significant differences in the duration of illness (*t* = −10.623, *P* < .001) and CPZ equivalents (*t* = −9.295, *P* < .001) between the TRS and non-TRS groups. Additionally, the TRS group exhibited significantly higher PANSS disorganization symptom scores compared to the non-TRS group (*t* = −3.884, *P* < .001), and comparisons for the other four dimensions (ie, positive, negative, affective symptoms, and resistance/activation) from the 5-factor model are provided in Table S1. There were also significant differences in the duration of illness (*t* = −10.623, *P* < .001) and CPZ equivalents (*t* = −9.295, *P* < .001) between the TRS and non-TRS groups. Finally, the mean framewise displacement (head motion) differed among the 3 groups (*F* = 3.208, *P* = .043), though the TRS group did not differ from non-TRS in this aspect.

**Table 1.** Demographic and Clinical Characteristics of the Final Participants

	HC (*n* = 64)	non-TRS (*n* = 76)	TRS (*n* = 48)	Statistics, *P*-value
Sex (male/female)	26/38	26/53	26/29	*χ* ^2^ = 2.861, *P* = .239^a^
Age (years)	24.94 ± 2.97	25.67 ± 7.41	30.19 ± 8.77	*F* = 9.604, *P* < .001^b^
Education (years)	16.03 ± 2.43	13.30 ± 2.90	13.10 ± 3.001	*F* = 21.619, *P* < .001^b^
Duration of illness (months)	-	22.03 ± 22.53	126.33 ± 80.94	*t* = −10.623, *P* < .001^c^
Chlorpromazine equivalents (mg/day)		256.12 ± 166.82	622.81 ± 272.84	*t* = −9.295, *P* < .001^c^
Positive and Negative Syndrome Scale (PANSS)				
Positive score	–	20.62 ± 5.17	21.21 ± 7.02	t = −0.538, *P* = .2592^c^
Negative score	–	17.71 ± 7.17	22.04 ± 6.84	*t* = −3.334, *P* = .001^c^
General psychopathology score	–	37.58 ± 9.04	40.10 ± 9.89	*t* = −1.461, *P* = .147^c^
Total score	–	75.98 ± 17.83	83.35 ± 19.90	*t* = −2.165, *P* = .032^c^
PANSS disorganization symptoms	–	16.93 ± 5.39	21.167 ± 6.66	*t* = −3.884, *P* < .001^c^
Mean framewise displacement (Jenkinson, mm)	0.068 ± 0.034	0.077 ± 0.041	0.089 ± 0.056	*F* = 3.208, *P* = .043^b^

Abbreviations: HC, healthy controls; non-TRS, non-treatment-resistant schizophrenia; TRS, treatment-resistant schizophrenia.

^a^Chi-square test.

^b^Analysis of Variance (ANOVA).

^c^Two-sample *t*-test.

### Spatiotemporal Pattern of the DMN

**Figure 2.**
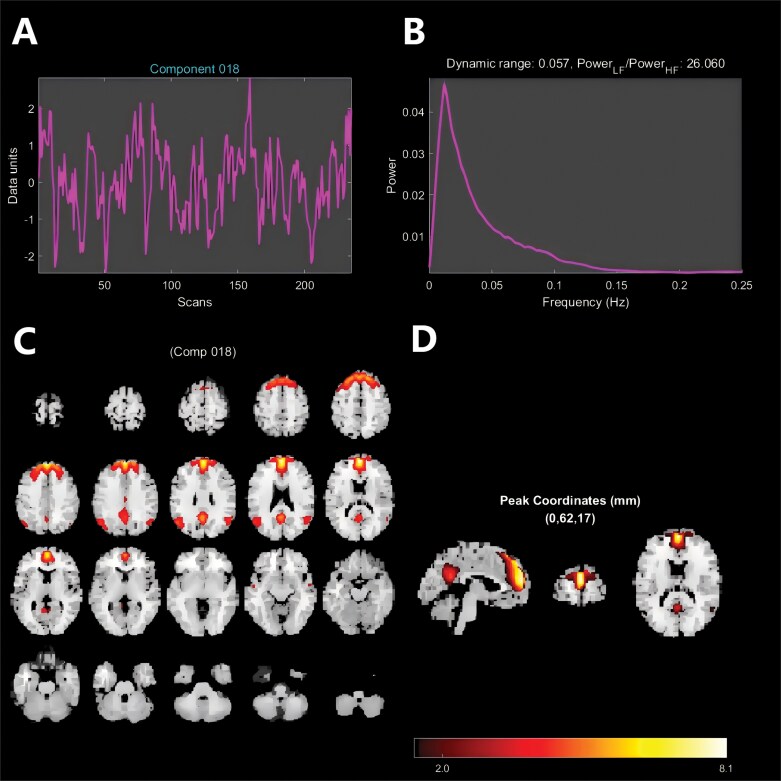
Group-Level Independent Component (IC18) Representing the Default Mode Network (DMN). (A) Mean time course of BOLD signal amplitude converted to *z*-scores. (B) Averaged power spectrum with dynamic range and low/high-frequency power ratio. (C) Axial slices showing the spatial distribution of the DMN component. (D) Orthogonal view at the peak voxel (coordinates: 0, 62, 17). The color bar (in web version) indicates the intensity of the *z*-value, which is derived from the correlation between the time series of each voxel and the mean time series of the whole network.

The group-level DMN spatial map and time course are shown in Figure 2. This component (reported as *z*-scores ≥2) includes the main nodes of the DMN including medial prefrontal cortex (MPFC) and posterior cingulate cortex, as well as bilateral inferior parietal lobules/angular gyrus, and bilateral medial temporal regions were also able to be detected albeit at a weaker level (*z*-scores ≥1). Spatial distribution of the DMN component is highly similar to previous studies.^10,66–68^

### Group Comparison of DMN Network-Level Metrics

**Figure 3.**
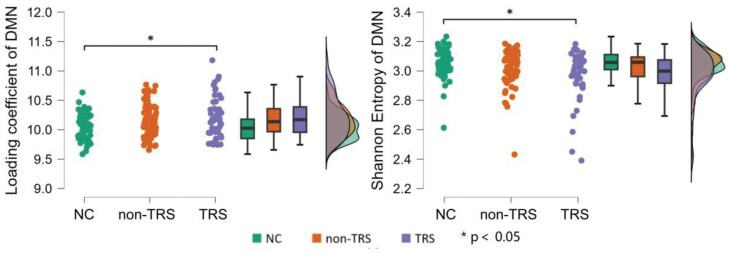
Group Comparisons of DMN Network-Level Metrics Among NC, non-TRS, and TRS group. Group comparisons were performed using Analysis of Covariance (ANCOVA), with age, sex, years of education, and mean framewise displacement as covariates. Tukey’s LSD test was conducted to test for group effects between any pair of the 3 groups. Significant differences between groups are indicated by *P < .05. DMN, default mode network; HC, healthy controls (first bar; in web version - green); non-TRS, non-treatment-resistant schizophrenia (second bar; in web version - orange); TRS, treatment-resistant schizophrenia (third bar; in web version - purple).

The ANCOVA results revealed a significant group effect on both DMN loading coefficient (*F* = 3.971, *P* = .021, Cohen’s *d* = 0.42) and Shannon entropy (*F* = 3.409, *P* = .035, Cohen’s *d* = 0.39), as shown in Table 2 and Figure 3. Specifically, the DMN’s loading coefficient was significantly higher in the TRS group compared to the HC group (*P* = .031), but there was no significant difference between the non-TRS and HC groups (*P* = .065), nor between the non-TRS and TRS groups (*p* = .794). DMN’s Shannon entropy was significantly lower in the TRS group compared to the HC group (*P* = .028), but did not differ between non-TRS and HC (*P* = .483) or between non-TRS and TRS (*P* = .156).

**Table 2.** Comparisons of the DMN Network-Level Metrics Across 3 Groups

Metrics	Standardized value (Mean ± SD)			ANCOVA		Post hoc(*P*_Tukey_)		
	HC	non-TRS	TRS	*F-*value	*P-*value	HC vs non-TRS	HC vs TRS	non-TRS vs TRS
Loading coefficients	10.031 ± 0.218	10.153 ± 0.271	10.214 ± 0.352	3.971	0.021^*^	0.065	0.031^*^	0.794
Shannon Entropy	3.053 ± 0.095	3.020 ± 0.118	2.970 ± 0.168	3.409	0.035^*^	0.483	0.028^*^	0.156

Abbreviations: ANCOVA, Analysis of Covariance; DMN, default mode network; HC, healthy controls; non-TRS, non-treatment-resistant schizophrenia; TRS, treatment-resistant schizophrenia.

Group comparisons after adjusting for age, sex, years of education, and mean framewise displacement. Significant differences between groups are indicated by ^*^(*P* < .05).

### Group Comparison of DMN Voxel-Level Metrics

**Figure 4.**
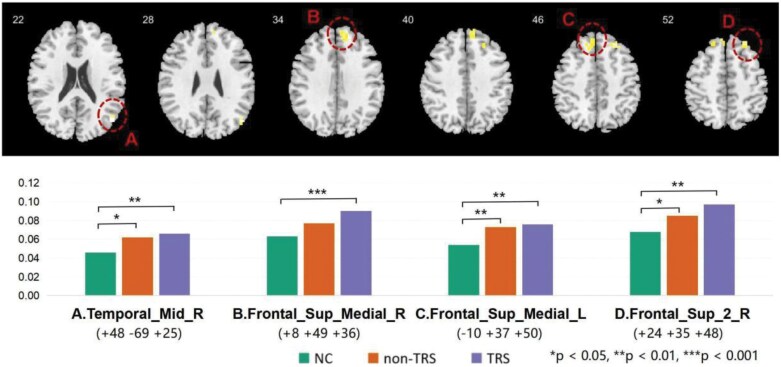
Brain Regions Within the DMN Show Group Differences in Network Homogeneity Across the 3 Groups. Significant differences between groups are indicated by asterisks, with *P < .05, **P < .01, and ***P < .001. DMN, default mode network; HC, healthy controls (first bar; in web version - green); non-TRS, non-treatment-resistant schizophrenia (second bar; in web version - orange); TRS, treatment-resistant schizophrenia (third bar; in web version - purple).

Figure 4 and Table 3 show significant differences in the NH values of the DMN across 3 groups, mainly in the bilateral superior medial frontal gyrus, the right superior frontal gyrus, as well as the right middle temporal gyrus. The mean NH values of these significant clusters were extracted for the post hoc tests (Figure 4 and Table S2). Results revealed that NH values of these regions in the TRS and non-TRS groups were significantly higher than in the HC group, generally following a pattern where TRS > non-TRS > HC, suggesting a gradient of increasing NH values within the DMN from the controls to non-TRS and TRS patients. We did not find any significant results for the voxel-wise Shannon entropy analysis.

**Table 3.** Regions with Significant Differences in Network Homogeneity (NH) of DMN in 3 Groups

AAL3 regions	MNI	*z*-Values of NH: Mean ± SD			ANCOVA		
	Coordinates	HC	Non-TRS	TRS	*F*-value	*P*-value	Cluster size
Temporal_Mid_R	48, −69, 25	0.046 ± 0.026	0.062 ± 0.033	0.066 ± 0.040	9.322	.000	13
Frontal_Sup_Medial_R	8, 49, 36	0.063 ± 0.024	0.077 ± 0.036	0.090 ± 0.050	7.370	.000	21
Frontal_Sup_Medial_L	−10, 37, 50	0.054 ± 0.019	0.073 ± 0.036	0.076 ± 0.041	7.077	.001	18
Frontal_Sup_2_R	24, 35, 48	0.068 ± 0.026	0.085 ± 0.040	0.097 ± 0.047	8.456	.000	20

Abbreviations: DMN, default mode network; AAL3, Automated Anatomical Labeling version 3; MNI, Montreal Neurological Institute; HC, healthy controls; non-TRS, non-treatment-resistant schizophrenia; TRS, treatment-resistant schizophrenia.

### Association Between DMN Metrics and Disorganization Symptoms

**Figure 5.**
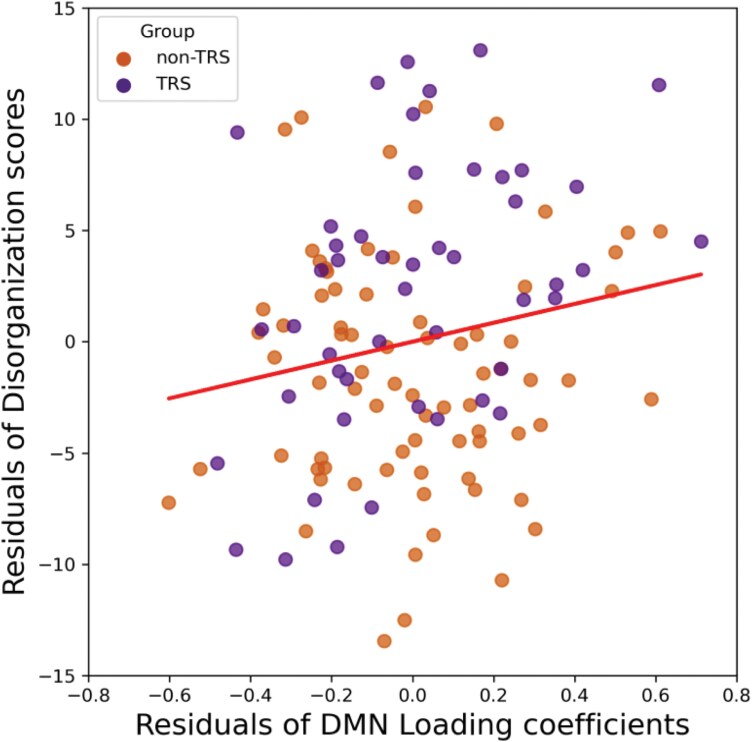
The Relationship Between the Loading Coefficient of the DMN and the Severity of Disorganization in the Patient Group (both TRS + non-TRS). DMN, default mode network; non-TRS, non-treatment-resistant schizophrenia (in web version - orange triangles); TRS, treatment-resistant schizophrenia (in web version - purple circles)

The multiple linear regression analysis indicated that the DMN loading coefficient was a significant predictor of disorganization symptoms (Β = 4.241, *P* = .038), with higher DMN loading associated with more severe disorganization symptoms across patients, as shown in Figure 5. However, DMN Shannon entropy did not show a significant relationship with disorganization (*P* > .05). Detailed analyses for the non-TRS, TRS, and combined patient groups are provided in Table S3. Notably, neither the loading coefficient nor Shannon entropy was found to significantly predict the severity of the other four symptom domains or cognitive scores (all *P* > .05), as detailed in Table S4. Furthermore, neither the DOI nor medication dosage showed any significant association with DMN metrics in either patient group.

### Exploratory Analysis of the Other RSNs

In addition to the DMN, we performed further exploratory analysis on other RSNs extracted from our dataset. Among the 20 ICs automatically estimated, we identified other 9 meaningful RSNs, including the sensorimotor network (SMN), auditory network (AN), language network, medial visual network (mVN), occipital VN (oVN), dorsal attention network, salience network(SN), left frontoparietal network (lFPN), and right FPN (rFPN), as shown in Figure S3. Using the same network-level analytical approach as for the DMN, we found an increased loading coefficient of SMN in TRS compared to non-TRS (*F* = 3.742, uncorrected *P* = .026, post hoc TRS > non-TRS, *P*_Tukey_ = .027), but the Shannon entropy of SMN did not differ from groups, and it was not correlated to disorganization. There were no significant group differences in other networks (Tables S6 and S7).

## Discussion

Investigating the role of DMN in TRS and disorganization using a 2-tiered spatiotemporal resting-state fMRI approach, we report excessive spatial prominence and temporal “hyperstability” of the DMN in TRS compared to healthy individuals. Importantly, non-TRS patients displayed an intermediate state of DMN disruption, suggesting that TRS may represent the extreme end of a “DMN disruption continuum”, rather than a distinct subtype. Voxel-wise analyses within DMN did not show significant differences between TRS and non-TRS groups, further supporting the idea that the changes we see in the DMN represent the pathophysiology of schizophrenia. The spatial excess of DMN was more pronounced in those with more severe disorganization indicating the shared pathophysiology between persistent disorganization and treatment resistance, as described in several prior studies.^40^ Thus, DMN dysfunction influences treatment responsiveness through a mechanistic pathway that is shared with the features of disorganization.

Our observations of DMN abnormalities in schizophrenia are consistent with several previous studies reporting DMN hyperconnectivity during task-free resting-state and attenuated deactivation of DMN during attention-demanding tasks, often linked to deficits in self-referential and introspective cognitive processes.^18–24,69–73^ We summarize the MRI studies that investigated DMN in TRS^74–80^ (Table S8, see Supplementary Materials for more details). Our study builds on this literature using multivariate gICA-derived loading coefficients and entropy to examine the DMN disruption. Unlike traditional univariate methods that focus on specific brain regions of DMN, here we captured the DMN as a cohesive whole, while simultaneously accounting for individual variability in its spatiotemporal expression across a heterogeneous population. Increased DMN loading coefficients in TRS suggest a proportionally greater contribution to the DMN in the presence of TRS, and possibly, higher than required engagement in DMN-mode of cognitive operations. This, combined with a loss of flexibility in the network’s temporal dynamics as revealed by lower Shannon entropy, is consistent with the systemic picture of triple network dysfunction reported elsewhere in schizophrenia.^14,81,82^

Voxel-wise network homogeneity (ie, intra-DMN connectivity) notably differed between patients and HC at several anterior DMN nodes, but showed no significant differences between TRS and non-TRS groups, akin to the network-level findings, emphasizing a continuum of DMN dysfunction across the schizophrenia spectrum. With DMN being spatially and temporally over-represented in TRS patients, an inability to adapt self-referential thinking and process external cognitive demands may occur. Interestingly, Wang and colleagues demonstrated that the DMN’s temporal variability may increase after 12 weeks of antipsychotic medication,^83^ indicating that in non-TRS patients, the relatively higher entropy might have occurred in response to treatment. While we discuss potential treatment-related changes in DMN variability, these changes are not directly measured in our study. The effect of first-line antipsychotics as well as clozapine on the spatiotemporal variability of DMN warrants further study using pharmaco-fMRI designs.

Our exploratory analysis of other ICA networks indicated higher SMN loading coefficients in TRS compared to non-TRS, while no significant differences were observed between either TRS or non-TRS and HC. This finding was restricted to increased spatial engagement without changes in the temporal patterns, and it did not correlate with disorganization symptoms. These results underscore the unique spatiotemporal involvement of DMN in TRS, which warrants further consideration. Margulies et al. identified a principal gradient of brain activity from unimodal to multimodal functions, with the multimodal DMN situated at the extreme end, allowing for the processing of cross-modal information unrelated to immediate sensory input.^84^ These findings support DMN’s critical role in higher-order cognitive processes and as a “default” state for human cognition.^10,11,66,67,85^ A combined disruption of the topography and temporal dynamics may preferentially affect networks that carry out multimodal functions that require abstract self-referential thinking, rather than unimodal regions.

DMN abnormalities have been strongly linked to deficits in executive functioning, disordered thought, and attentional disruptions, the key features of disorganization symptoms.^13^ Our analysis revealed that the DMN loading coefficient was higher in patients with more severe disorganization throughout the entire patient group, regardless of treatment response. This is consistent with our hypothesis that DMN may provide a mechanistic substrate for linguistic cognition (meaning generation), which when disrupted could affect 2 key functions of language—thought and communication, resulting in the psychopathology of disorganization.^86^ Further support for this claim comes from our exploratory analysis (see Supplementary Materials) showing that the DMN loading coefficient specifically relates to the burden of disorganization, but no other symptoms or cognitive performance. Thus, the spatial prominence of DMN connectivity at rest specifically marks disorganization in schizophrenia. In our sample, we did not collect data on speech outputs or social cognition (such as theory of mind tasks) that contribute to disorganization.^87,88^ Therefore, our interpretations remain speculative. Future studies should include such assessments to directly test the proposed link between DMN abnormalities and disorganization-related cognitive processes.

Our study has other notable limitations that warrant discussion. First, although the 2 patient groups significantly differed in terms of CPZ daily equivalents, which is in line with prior studies on TRS, this difference did not correlate significantly with DMN metrics. While we were unable to collect the detailed information about cumulative antipsychotic exposure, TRS patients generally have higher cumulative antipsychotic exposure than non-TRS patients. Second, except for positive symptoms, TRS subjects in our study showed higher scores on positive and general subscales of the PANSS classic 3-factor, as well as higher scores on negative, disorganization, resistance/activation, and lower scores on affective dimensions of the PANSS 5-factor symptom models, indicating more severe symptoms in the TRS group, which is a hallmark of TRS itself. Therefore, the potential influence of symptom chronicity cannot be entirely excluded. These potential confounders could not be fully accounted for and warrant further investigation in future longitudinal studies with more precise drug exposure to observe the progression of DMN changes. We defined disorganization domain exclusively on the basis of PANSS items. While this approach is widely used and shown to be reliable across studies, further research incorporating detailed and structured scales focused on disorganization, like the Thought and Language Disorder scale,^89^ would be valuable for a more comprehensive evaluation. Another potential limitation lies in our study’s focus on DMN disruption without directly assessing temporal fragmentation,^90,91^ which reflects an impaired ability to experience time as a continuous flow and may manifest as reduced temporal variability in the DMN. Future research could incorporate such scales to better capture the relationship between DMN dysfunction and fragmented time perception.

## Conclusions

In summary, TRS represents the more severe end of a continuum of dysfunction of the DMN in schizophrenia, characterized by greater spatiotemporal representation of this intrinsic network. The spatial prominence of the DMN is uniquely linked to symptoms of disorganization, while both spatial and temporal aberrations become more pronounced with refractoriness to first-line treatments. Therapeutic strategies that target the DMN and its neural substrates, especially those that improve its spatiotemporal patterns and address disorganization, may potentially reduce or reverse treatment resistance in schizophrenia.

## Supplementary Material

sbaf018_suppl_Supplementary_Figures_S1-S3_Tables_S1-S8

## Data Availability

The datasets generated and/or analyzed during the current study are not publicly available as they contain patients’ personal information that cannot be publicly shared due to the hospital policy, but are available from the corresponding author on reasonable request.
