# Supplementary material for: Default Mode Network, Disorganization, and Treatment-Resistant Schizophrenia
Source: Schizophr Bull. 2025 Mar 3;52(1):sbaf018. doi: 10.1093/schbul/sbaf018 (PMC12809848; doi:10.1093/schbul/sbaf018)
Supplement: sbaf018_suppl_Supplementary_Figures_S1-S3_Tables_S1-S8 [file sbaf018_suppl_supplementary_figures_s1-s3_tables_s1-s8.docx]

**Supplemental Materials**

**[1. Participant Exclusion and Final Sample Composition 2](#_Toc2693)**

**[2. Imaging Data preprocessing 2](#_Toc7695)**

**[3. Spatial group independent component analysis (gICA) work flow 4](#_Toc10290)**

**[4. DMN Mask Generation 4](#_Toc29946)**

**[5. Extracting loading coefficients 5](#_Toc24765)**

**[6. Comparison of PANSS five main dimensions for non-TRS and TRS groups 6](#_Toc11279)**

**[7. Post hoc tests of Network Homogeneity (NH) among three groups 6](#_Toc20346)**

**[8. Regression analysis between DMN metrics and disorganization domain 7](#_Toc392)**

**[9. Regression analysis between DMN metrics and other four symptom domains 7](#_Toc20770)**

**[10. Effect of cognitive performance on DMN integrity 8](#_Toc16634)**

**[11. Effect of first-episode patients in the non-TRS group 9](#_Toc26030)**

**[12. Network-level analysis of other RSNs 10](#_Toc10826)**

**[13. A selected review of MRI studies of default mode network abnormalities in treatment-resistant schizophrenia 14](#_Toc22011)**

**[References 17](#_Toc28998)**

# Participant Exclusion and Final Sample Composition

A total of 211 participants were initially recruited for the current study, including 66 healthy controls (HC), 86 individuals with non-treatment-resistant schizophrenia (non-TRS), and 59 individuals with treatment-resistant schizophrenia (TRS). Following recruitment, 23 participants were excluded for various reasons. Specifically, four participants in the non-TRS group and two in the TRS group were excluded because they were unable to complete the entire MRI scan. Additionally, visual inspection of image quality led to the exclusion of two participants each from the HC, non-TRS, and TRS groups due to poor image quality. Furthermore, participants with excessive head motion, defined by a Jenkinson framewise displacement (FD) greater than 0.2, resulted in the exclusion of four participants in the non-TRS group and six in the TRS group. After these exclusions, 188 participants were included in the final analysis: 64 HC, 76 individuals with non-TRS, and 48 individuals with TRS.

# Imaging Data preprocessing

Results included in this manuscript come from preprocessing performed using

*fMRIPrep 24.0.0* (RRID:SCR_016216)^1^, which is based on *Nipype 1.8.6* (RRID:SCR_002502). The following boilerplate text was automatically generated by fMRIPrep, with removing the unused details. It is released under the CC0 license.

**Anatomical data preprocessing:** The T1w image of each subject was corrected for intensity non-uniformity (INU) with N4BiasFieldCorrection^2^, distributed with ANTs 2.5.1 (RRID:SCR_004757)^3^, and used as T1w-reference throughout the workflow. The T1w-reference was then skull-stripped with a Nipype implementation of the antsBrainExtraction.sh workflow (from ANTs), using OASIS30ANTs as target template. Brain tissue segmentation of cerebrospinal fluid (CSF), white-matter (WM) and gray-matter (GM) was performed on the brainextracted T1w using fast (FSL 6.0.7.11, RRID:SCR_002823)^4^. Brain surfaces were reconstructed using recon-all (FreeSurfer 7.3.2, RRID:SCR_001847)^5^, and the brain mask estimated previously was refined with a custom variation of the method to reconcile ANTs-derived and FreeSurfer-derived segmentations of the cortical gray-matter of Mindboggle (RRID:SCR_002438)^6^. Volume-based spatial normalization to two standard spaces (MNI152NLin6Asym, MNI152NLin2009cAsym) was performed through nonlinear registration with antsRegistration (ANTs 2.5.1), using brain-extracted versions of both T1w reference and the T1w template. The following templates were were selected for

spatial normalization and accessed with *TemplateFlow* (24.2.0)^7^: *FSL’s MNI ICBM 152 non-linear 6th Generation Asymmetric Average Brain Stereotaxic Registration Model* (RRID:SCR_002823; TemplateFlow ID: MNI152NLin6Asym), *ICBM 152 Nonlinear Asymmetrical template version 2009c* (RRID:SCR_008796; TemplateFlow

ID: MNI152NLin2009cAsym).

**Functional data preprocessing:** For resting-state fMRI BOLD image of each subject, the following preprocessing steps wereperformed. First, a reference volume was generated, using a custom methodology of *fMRIPrep,* for use in head motion correction. Head motion parameters with respect to the BOLD reference (transformation matrices, and six corresponding rotation and translation parameters) are estimated before any spatiotemporal filtering using mcflirt (FSL 6.0.7.11)^8^. Slice time correction were performed prior to other signal resampling processes using 3dTShift (AFNI), and all slices were realigned in time to the middle of each TR. The BOLD reference was then co-registered to the T1w reference using bbregister (FreeSurfer) which implements boundary-based registration^9^. Co-registration was configured with six degrees of freedom. All resamplings were performed with a single interpolation step by composing all the pertinent transformations (i.e., head-motion transform matrices, and co-registrations to anatomical and output spaces). No gridded (volumetric) resampling of BOLD image was performed to preserve the original voxel size. Additionally, the following confounding time-series were calculated and extracted based on the *preprocessed BOLD data* and be used for further nuisance regression steps where needed : head-motion estimates, WM, and CSF region-wise signals. The head-motion estimates included Friston’s 24-parameter model, which comprises 6 motion parameters, 6 temporal derivatives, and 12 squared terms, accounting for translational (X, Y, Z) and rotational (pitch, roll, yaw) movements, along with their temporal derivatives and squared terms to mitigate motion-related artifacts^10^. The WM and CSF signals were calculated as the average signals within anatomically-derived, eroded WM and CSF masks, respectively.

The mean FD for each subject was also computed using the formulations following Jenkinson (relative root mean square displacement between affines)^8^, and the subjects with mean FD greater than 0.2 were removed.

# Spatial group independent component analysis (gICA) work flow

**Figure S1. Group Independent Component Analysis (gICA) Workflow for Source-based DMN Extraction**

**
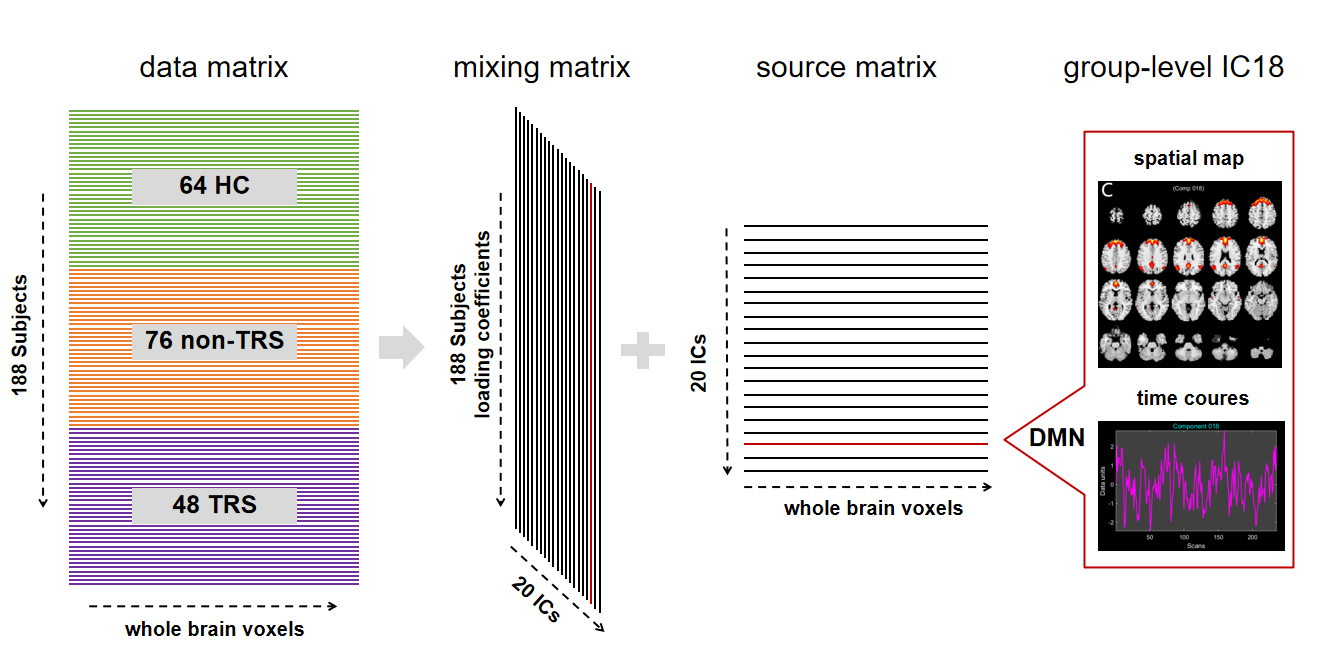
**

Notes: HC, healthy controls; non-TRS, non-treatment-resistant schizophrenia; TRS, treatment-resistant schizophrenia; DMN, default mode network; IC, independent component

# DMN Mask Generation

To generate a DMN mask for the voxel-level analysis, one-sample t-tests were performed on the subject-specific DMN independent component (IC) maps within each group to create group-level statistical maps. The resulting statistical maps were thresholded at a voxel-level significance of p<0.01 (FWE-corrected) with a minimum cluster size of 50 voxels. These thresholded maps were then combined across the three groups to produce an explicit DMN mask used for subsequent within DMN voxel-level analyses.

**Figure S2. The spatial display of group-level** **DMN statistical maps and DMN binary mask for voxel-wise analysis**


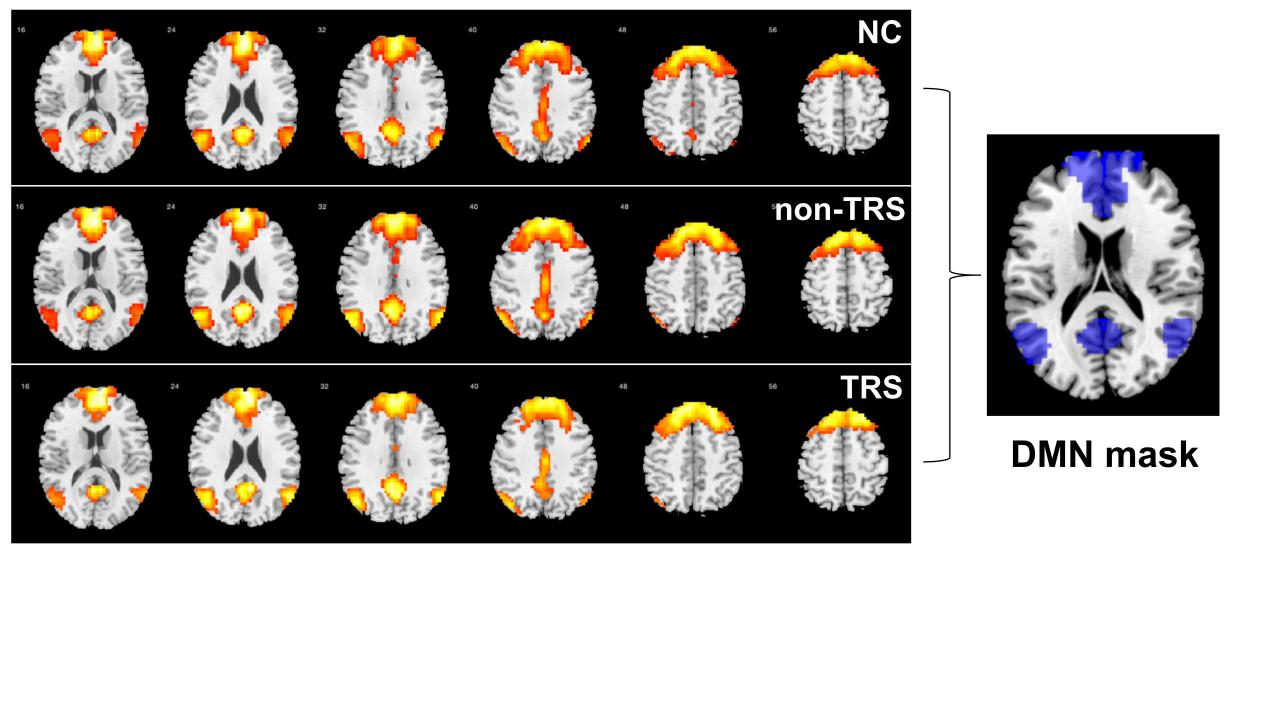


Notes: DMN, default mode network; HC, healthy controls; non-TRS, non-treatment-resistant schizophrenia; TRS, treatment-resistant schizophrenia. Group-level DMN statistical maps (left) were calculated by one-sample t-tests on the subject-specific DMN independent component (IC) maps in each group separately wiht a thresholded at a voxel-level FWE-corrected of p<0.01 and cluster size >50 voxels); the binary DMN mask (right) were generated by combining the thresholded binary three group-level DMN statistical maps .

# Extracting loading coefficients

These were directly calculated using the GIFT toolbox as part of the standard Group ICA process. Specifically, GIFT performs a back-reconstruction step after identifying the group-level independent components, estimating the contribution of each subject to these components. The subject-specific loading coefficients are output as a NIfTI file (ica_subject_loadings.nii) by GIFT. In our analysis, this file has a structure of 188 x 1 x 20, where 188 represents the number of subjects, 1 indicates a single session per subject, and 20 corresponds to the number of independent components extracted by ICA. The loading coefficients in this file reflect each subject’s contribution to the 20 spatially independent brain networks identified during the ICA. We then wrote a simple MATLAB script that used niftiread function to extract these coefficients from the NIfTI file, and no alternative methods were employed for this calculation. The exact code used for this extraction is available here: [https://github.com/huanhuang1988/gICA_loading_coefficient_extraction](https://github.com/huanhuang1988/gICA_loading_coefficient_extraction.).

# Comparison of PANSS five main dimensions for non-TRS and TRS groups

We also compared the five main dimensions proposed by Shafer and Dazzi in a recent meta-analysis on the PANSS factor structure^11^: Positive Symptoms, Negative Symptoms, Disorganization, Affect (Depression/Anxiety) and Resistance/Activation. The two groups showed no significant difference in positive symptoms (p = 0.680), but the TRS group had significantly higher scores on the other dimensions. Specifically, TRS patients had significantly elevated scores in negative symptoms (p = 0.003), disorganization symptoms (p < 0.001), resistance/activation (p = 0.009), and lower scores in affect (depression/anxiety) (p = 0.007). These results indicate that the TRS group exhibited more severe negative, disorganization, and activation symptoms, which are typical characteristics of TRS, while the non-TRS group displayed higher affective symptoms.

| **Table S1. PANSS five main dimensions** **for two patient groups** | | | |
| --- | --- | --- | --- |
|  | **non-TRS(n=76)** | **TRS(n=48)** | **t value, p value** |
| PANSS dimensions |  |  |  |
| Positive symptoms | 19.88±5.21 | 19.44±6.67 | t=0.414, p=0.680 |
| Negative symptoms | 18.24±7.20 | 22.10±6.73 | t=-2.988, p=0.003 |
| Disorganization symptoms | 16.93±5.39 | 21.17±6.66 | t=-3.884, p<0.001 |
| Affect (Depression/Anxiety) | 12.61±4.80 | 10.40±3.52 | t=2.753, p=0.007 |
| Resistance/Activation | 8.25±3.55 | 10.25±4.83 | t=-2.652, p=0.009 |
| Note: PANSS, Positive and negative syndrome scale; non-TRS, non-treatment-resistant schizophrenia; TRS, treatment-resistant schizophrenia. | | | |

# Post hoc tests of Network Homogeneity (NH) among three groups

| **Table S2. Post-hoc pairwise comparisons of DMN Network Homogeneity** | | | |
| --- | --- | --- | --- |
| **AAL3 regions** | **Post poc comparison P**Tukey | | |
|  | **HC vs. non-TRS** | **HC vs. TRS** | **non-TRS vs. TRS** |
| Temporal_Mid_R | 0.019 | 0.000 | 0.212 |
| Frontal_Sup_Medial_R | 0.002 | 0.000 | 0.435 |
| Frontal_Sup_Medial_L | 0.003 | 0.003 | 0.782 |
| Frontal_Sup_2_R | 0.010 | 0.000 | 0.177 |
| Notes: DMN, default mode network; AAL3, Automated Anatomical Labeling version 3; HC, healthy controls; non-TRS, non-treatment-resistant schizophrenia; TRS, treatment-resistant schizophrenia. | | | |

# Regression analysis between DMN metrics and disorganization domain

The multiple linear regression analysis indicated that the DMN loading coefficient was a significant predictor of disorganization symptoms (β = 4.241, p = 0.038), with higher DMN loading associated with more severe disorganization symptoms across the full patient group (β = 4.241, p = 0.038), and also in TRS group alone (β = 8.345, p = 0.012).

| **Table S3. Multiple regression analysis of disorganization in the patient groups** | | | |
| --- | --- | --- | --- |
| Independent variables | dependent variables: Disorganization | | |
|  | **non-TRS+TRS** | **non-TRS** | **TRS** |
|  | F=4.006, p=0.001 | F=2.266, p=0.047 | F=3.727, p=0.005 |
|  | unstandardized β  (p value) | unstandardized β  (p value) | unstandardized β  (p value) |
| age | -0.007(0.915) | -0.155（0.082） | -0.043(0.691) |
| gender | **-2.333(0.048)** | -1.562（0.276） | -2.129(0.247) |
| education | **-0.707(0.001)** | -0.325（0.143） | **-0.929(0.005)** |
| mean framewise displacement | -1.517(0.905) | 1.959（0.908） | -4.560(0.809) |
| DMN loading coefficient | **4.241(0.038)** | -0.134（0.955） | **8.345(0.012)** |
| DMN Shannon entropy | 2.704(0.493) | 8.206（0.122） | 2.800(0.603) |
| Notes:DMN, default mode network; non-TRS, non-treatment-resistant schizophrenia; TRS, treatment-resistant schizophrenia. | | | |

# Regression analysis between DMN metrics and other four symptom domains

We conducted multiple regression analyses to explore the relationship between DMN metrics (both loading coefficient and Shannon entropy) and the other four symptom domains in the full patient group. These analyses included age, sex, years of education, and mean FD as covariates. The results showed that neither the loading coefficient nor the Shannon entropy significantly predicted the severity of these additional symptom domains (all p > 0.05).

| **Table S4. Multiple regression analysis of other symptoms in the patient groups** | | | |  |
| --- | --- | --- | --- | --- |
| **Symptoms** | **regression model** | **DMN loading coefficient unstandardized β (p value)** | **DMN Shannon entropy unstandardized β (p value)** | |
| Positive symptoms | F=0.493, p=0.813 | 2.703(0.188) | -2.289(0.564) |  |
| Negative symptoms | F=1.935, p=0.081 | 4.090(0.099) | 3.799(0.431) |  |
| Affect (Depression/Anxiety) | F=3.897, p=0.001 | -1.286(0.1378) | -1.619(0.567) |  |
| Resistance/Activation | F=1.058, p=0.392 | -0.543(0.710) | -0.416(0.883) |  |
| Notes:DMN, default mode network; non-TRS, non-treatment-resistant schizophrenia; TRS, treatment-resistant schizophrenia. | | | |  |

# Effect of cognitive performance on DMN integrity

Disorganization shares notable variance with cognitive function and this symptom domain is often termed “cognitive disorganization” in previous studies, underscoring the significant overlap. This overlap makes it difficult to fully disentangle cognitive function from this symptom domains based solely on PANSS score. Regressing out cognition from disorganization results in a variable that is not reflective of the real-world variance i.e., creating a model where disorganization varies when cognition is held constant. Instead, we examined the relevance of cognitive performance per se on DMN integrity in a subset of patients for whom we collected Brief Assessment of Cognition in Schizophrenia (BACS) [165 participants (64 NC, 60 non-TRS, and 41 TRS)]. We calculated a composite BACS score as a measure of overall cognitive performance and conducted a similar regression analysis to examine if it can be predicted by DMN loading coefficient and/or DMN Shannon entropy, with age, gender, education, and mean FD as covariates. The results show that neither DMN loading coefficient (p = 0.129) nor DMN Shannon entropy (p = 0.128) emerged as significant predictors of cognitive performance. This suggests that, in our sample, network-level DMN metrics were not directly associated with cognitive performance, as measured by BACS. In summary, our findings indicate that although DMN loading coefficient correlates with disorganization symptoms, it does not seem to predict general cognitive function in our patient sample. This may further support the distinction between cognitive and disorganization domains, even though they exhibit substantial overlap.

# Effect of first-episode patients in the non-TRS group

We stratified the n=34 first-episode psychosis (FEP) patients from multi-episode psychosis (MEP) among the non-TRS. We then performed additional analyses by dividing the 188 participants into four groups (HC = 64, FEP = 34, MEP-non-TRS = 42, TRS = 48), and the results are shown in the Table below. We continue to see an omnibus group effect, albeit marginal now, for loading coefficient (F = 2.633, p = 0.05), and for Shannon entropy (F = 2.797, p = 0.042), with post-hoc analysis still supporting HC>TRS for both measures. However, there were no significant differences between the two non-TRS groups: MEP and FEP groups and between FEP with HC or TRS in either measure. We do note that for Shannon entropy, the effect size of MEP vs TRS comparison (d=0.46) is higher than that for FEP vs TRS (d=0.22), indicating that in the non-TRS patients with longer exposure to antipsychotics (MEP sub-group), a relatively higher entropy (closer to the healthy group) might have occurred in response to prior treatment. Nevertheless, we did not see a significant difference between MEP and FEP (p=0.6). This supports our approach to consider the two subgroups of non-TRS in one group as non-TRS. This decision allowed us to concentrate on treatment resistance and its impact on DMN dysfunction, which is

central to our research question.

| **Table S5. Comparisons of the DMN network-level metrics across four groups** | | | | | | | | | | | | | | |
| --- | --- | --- | --- | --- | --- | --- | --- | --- | --- | --- | --- | --- | --- | --- |
| **metrics** | **standardized value(Mean±SD)** | | | |  | **ANCOVA** | |  | **Post Hoc (P_Tukey_)** | | | | | |
|  | **HC** | **FEP** | **MEP** | **TRS** |  | **F** | **p** |  | **HC vs. FEP** | **HC vs. MEP** | **HC vs. TRS** | **FEP vs. MEP** | **FEP vs. TRS** | **MEP vs. TRS** |
| Loading coefficients | 10.03  ±0.22 | 10.19  ±0.30 | 10.13  ±0.25 | 10.21  ±0.35 |  | 2.63 | **0.05** |  | 0.28 | 0.23 | 0.06 | 1.00 | 0.95 | 0.94 |
| Shannon Entropy | 3.05  ±0.10 | 3.00  ±0.13 | 3.03  ±0.11 | 2.97  ±0.17 |  | 2.79 | **0.04** |  | 0.35 | 0.97 | **0.04** | 0.60 | 0.80 | 0.12 |
| Notes:DMN, default mode network; HC, healthy controls; FEP, non-treatment-resistant schizophrenia, first-episode; MEP, non-treatment-resistant schizophrenia, multi-episode; ; TRS, treatment-resistant schizophrenia; ANCOVA, Analysis of Covariance; group comparisons after adjusting for age, sex, years of education, and mean framewise displacement | | | | | | | | | | | | | | |

# Network-level analysis of other RSNs

We conducted an exploratory analysis by selecting the other RSNs simultaneously extracted by ICA, including the sensorimotor network (SMN), auditory network (AN), language network (LN), medial visual network (mVN), occipital VN (oVN), dorsal attention network (DAN), salience network(SN), left frontoparietal network (FPN), and right FPN. Using the same network-level analytical approach as for the DMN, we found increased loading coefficient of SMN in TRS compared to non-TRS (F = 3.742, p = 0.026, post hoc indicates TRS > non-TRS, P_Tukey_ = 0.027), but the Shannon entropy of SMN did not differ from groups, also it was not correlated to disorganization. There were no significant group differences in other networks.

**Figure S3. The nine group-level RSNs Independent Components**


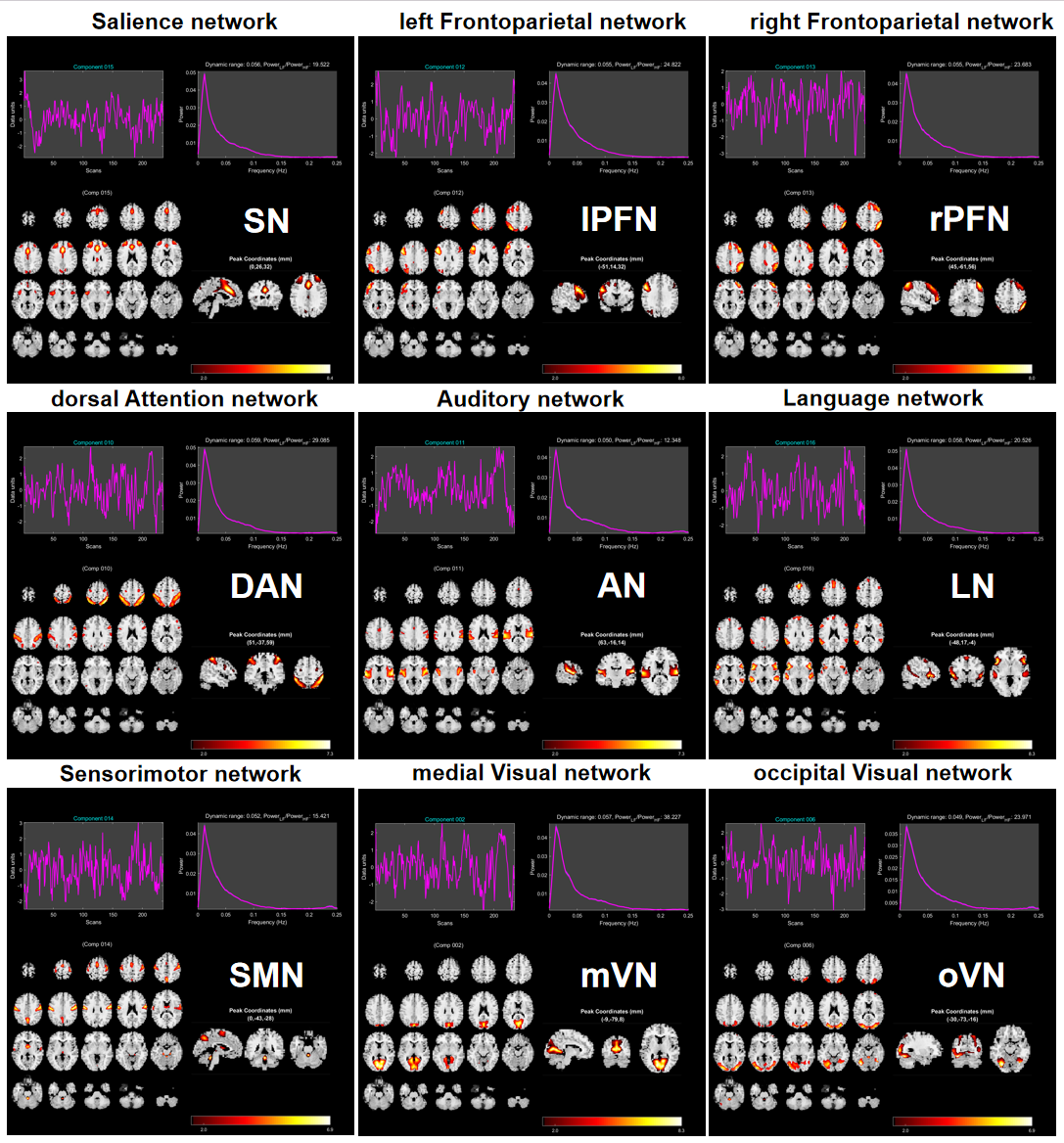


Notes: RSN, resting-state network; Mean time course converted to z-scores, showing BOLD signal fluctuations; Averaged power spectrum with dynamic range and low/high-frequency power ratio; Axial slices showing the spatial distribution of the RSN component; Orthogonal view at the peak voxel. The color bar indicates the intensity of the z-value, which derived from the correlation between the time-series of each voxel and the mean time-series of the whole network.

| **Table S6. ANCOVA of the RSNs loading coefficients across three groups** | | | | | | | |
| --- | --- | --- | --- | --- | --- | --- | --- |
| **RSNs** | **IC number** | **Log-transformed value (Mean±SD)** | | |  | **ANCOVA** | |
|  |  | **HC** | **non-TRS** | **TRS** |  | **F value** | **p Value** |
| Salience network (SN) | IC15 | 10.119±0.235 | 10.181±0.302 | 10.248±0.361 |  | 0.014 | 0.986 |
| left Frontoparietal network (lFPN) | IC12 | 10.141±0.245 | 10.221±0.251 | 10.243±0.348 |  | 0.185 | 0.831 |
| right Frontoparietal network (rFPN) | IC13 | 10.118±0.291 | 10.183±0.259 | 10.280±0.381 |  | 0.410 | 0.664 |
| dorsal Attention network (DAN) | IC10 | 10.283±0.343 | 10.350±0.365 | 10.387±0.339 |  | 0.238 | 0.789 |
| Auditory network (AN) | IC11 | 10.182±0.240 | 10.235±0.279 | 10.363±0.325 |  | 1.192 | 0.306 |
| Language network (LN) | IC16 | 10.218±0.312 | 10.286±0.333 | 10.343±0.368 |  | 0.157 | 0.885 |
| Sensorimotor network (SMN) | IC14 | 10.430±0.274 | 10.398±0.255 | 10.549±0.291 |  | 3.742 | **0.026** |
| medial Visual network (mVN) | IC02 | 10.334±0.417 | 10.412±0.429 | 10.571±0.463 |  | 0.714 | 0.491 |
| occipital Visual network (oVN) | IC06 | 10.322±0.348 | 10.386±0.372 | 10.526±0.350 |  | 0.964 | 0.383 |
| Notes: RSN, resting-state network; IC, independent component; HC, healthy controls; non-TRS, non-treatment-resistant schizophrenia; TRS, treatment-resistant schizophrenia; ANCOVA, Analysis of Covariance; group comparisons after adjusting for age, sex, years of education, and mean framewise displacement. | | | | | | | |

| **Table S7. ANCOVA of the RSNs Shannon entropy across three groups** | | | | | | | |
| --- | --- | --- | --- | --- | --- | --- | --- |
| **RSNs** | **IC number** | **entropy value (Mean±SD)** | | |  | **ANCOVA** | |
|  |  | **HC** | **non-TRS** | **TRS** |  | **F value** | **p Value** |
| Salience network (SN) | IC15 | 2.984±0.145 | 2.982±0.200 | 2.906±0.238 |  | 1.391 | 0.251 |
| left Frontoparietal network (lFPN) | IC12 | 2.998±0.148 | 3.017±0.123 | 3.016±0.128 |  | 0.280 | 0.756 |
| right Frontoparietal network (rFPN) | IC13 | 3.019±0.119 | 3.021±0.122 | 2.966±0.209 |  | 1.626 | 0.200 |
| dorsal Attention network (DAN) | IC10 | 3.041±0.114 | 2.978±0.156 | 2.946±0.166 |  | 1.997 | 0.139 |
| Auditory network (AN) | IC11 | 3.002±0.119 | 2.998±0.117 | 2.973±0.194 |  | 0.441 | 0.664 |
| Language network (LN) | IC16 | 3.022±0.092 | 3.032±0.092 | 2.999±0.157 |  | 0.543 | 0.582 |
| Sensorimotor network (SMN) | IC14 | 2.954±0.184 | 2.966±0.162 | 2.935±0.219 |  | 0.299 | 0.742 |
| medial Visual network (mVN) | IC02 | 2.949±0.149 | 2.952±0.167 | 2.888±0.233 |  | 1.994 | 0.139 |
| occipital Visual network (oVN) | IC06 | 2.955±0.170 | 2.946±0.175 | 2.867±0.259 |  | 2.084 | 0.127 |
| Notes: RSN, resting-state network; IC, independent component; HC, healthy controls; non-TRS, non-treatment-resistant schizophrenia; TRS, treatment-resistant schizophrenia; ANCOVA, Analysis of Covariance; group comparisons after adjusting for age, sex, years of education, and mean framewise displacement. | | | | | | | |

# A selected review of MRI studies of default mode network abnormalities in treatment-resistant schizophrenia

Systematic searches of relevant articles were conducted from the electronic database, including Ovid Medline, Embase, Pubmed, PsychINFO, and Web of Science, from inception to 05 September 2024. Studies that were published in English in a peer-reviewed journal with study samples including schizophrenia, schizoaffective disorder, and schizophreniform disorders according to Diagnostic and Statistical Manual of Mental Health (DSM) or International Classification of Diseases (ICD) criteria, having an operationalized definition of treatment resistance. All cross-sectional and longitudinal studies were included. Conference abstracts, theses, and editorials were excluded. References from other review articles were examined for relevant studies.An electronic database search was conducted using the following syntax as search terms:

(“treatment-resistant” OR “treatment resistant” OR “treatment-resistance” OR “treatment resistance” OR “nonresponder” OR“non-responder” OR “refractory” OR “medication-resistant” OR “antipsychotic resistant” OR “ultratreatment-resistant” OR “clozapine-resistant”) AND (“schizophrenia” OR “schizoaffective” OR “schizophreniform” OR “psychosis” OR “TRS” OR “UTRS”) AND (“default mode network” OR “default network” OR “DMN” OR “posterior cingulate cortex” OR “PCC” OR “medial prefrontal cortex” OR “mPFC” OR “MPFC”) AND (“magnetic resonance imaging” OR “MRI” OR “diffusion tensor imaging” OR “DTI” OR “diffusion weighted imaging” OR “DWI” OR “functional magnetic resonance imaging” OR “fMRI” OR “structural magnetic resonance imaging” OR “sMRI” OR “diffusional magnetic resonance imaging” OR “dMRI"OR “voxel-based morphometry” OR “VBM” OR “surface-based morphometry” OR “SBM” OR “functional connectivity” OR “structural connectivity” OR “seed-based analysis” OR “group independent component analysis” OR “gICA” OR “group ICA”)

| **Table S8. A Selected Review of MRI studies on DMN and TRS**^12-18^ | | | | | |
| --- | --- | --- | --- | --- | --- |
| **ID** | **Study** | **Design** | **Indices** | **sample** | **Main DMN-related findings** |
| 1 | Crisp CM et al., 2024 | Longitudinal | MPFC activity and glutamate levels changes | 15 TRS, 35 non-TRS | MPFC activity deteriorated in TRS; MPFC dysfunction and uncoupling with glutamate may predict resistance |
| 2 | Kitajima K et al., 2023 | Cross-sectional | Local gyrification index (LGI) and surface area (SA) analysis | 41 TRS, 20 non-TRS, 24 HC | TRS patients had reduced LGI and SA in the left medial parietal cortex; SA correlated with negative symptoms and clozapine levels |
| 3 | Huang H et al., 2018 | Longitudinal | global functional connectivity density (gFCD) changes after treatment | 21 TRS, 21 schizophrenia, 23 HC | TRS group showed increased gFCD in dMPFC, vMPFC, and left Pcu after 4-week modified electroconvulsive therapy (MECT) |
| 4 | Potvin S et al., 2015 | Cross-sectional | Emotion processing task activation | 22 TRS, 24 non-TRS, 39 HC | TRS showed hyperactivation in dMPFC and cerebellum (emotion images), and cingulate gyrus (neutral images) |
| 5 | Ahmed M et al., 2015 | Longitudinal | progressive change of brain volume and cortical thickness | 33 TRS, 31 HC | Greater volume reductions in MPFC and periventricular area in TRS; cortical thinning linked to less symptom improvement |
| 6 | Alonso-Solís A et al., 2015 | Cross-sectional | Seed-based correlation analysis of DMN hubs and subsystems | 19 resistant AVH, 14 non-AVH, 20 HC | Higher functional connectivity (FC) between dMPFC and salience-related regions, and lower FC between vMPFC and anterior cingulate cortex in resistant AVH patients |
| 7 | Kubera KM et al., 2014 | Cross-sectional | source-based morphometry and loading coefficients | 10 persistant AVH and 10 non-AVH | persistant AVH showed reduced structural covariance of medial and inferior frontal, insular and bilateral temporal gray matter volume |
| Notes: MRI, magnetic resonance imaging; DMN, default mode network; TRS, treatment-resistant schizophrenia; non-TRS, non-treatment-resistant schizophrenia; HC, healthy controls; MPFC, medial prefrontal cortex; AVH, auditory verbal hallucinations | | | | | |
|  |  |  |  |  |  |

**References**

**1.** Esteban O, Markiewicz CJ, Blair RW, et al. fMRIPrep: a robust preprocessing pipeline for functional MRI. *Nat Methods* Jan 2019;16(1):111-116.

**2.** Tustison NJ, Avants BB, Cook PA, Zheng Y, Egan A, Yushkevich PA, Gee JC. N4ITK: improved N3 bias correction. *IEEE Trans Med Imaging* Jun 2010;29(6):1310-1320.

**3.** Avants BB, Epstein CL, Grossman M, Gee JC. Symmetric diffeomorphic image registration with cross-correlation: evaluating automated labeling of elderly and neurodegenerative brain. *Med Image Anal* Feb 2008;12(1):26-41.

**4.** Zhang Y, Brady M, Smith S. Segmentation of brain MR images through a hidden Markov random field model and the expectation-maximization algorithm. *IEEE Trans Med Imaging* Jan 2001;20(1):45-57.

**5.** Dale AM, Fischl B, Sereno MI. Cortical surface-based analysis. I. Segmentation and surface reconstruction. *Neuroimage* Feb 1999;9(2):179-194.

**6.** Klein A, Ghosh SS, Bao FS, et al. Mindboggling morphometry of human brains. *PLoS Comput Biol* Feb 2017;13(2):e1005350.

**7.** Ciric R, Thompson WH, Lorenz R, et al. TemplateFlow: FAIR-sharing of multi-scale, multi-species brain models. *Nat Methods* Dec 2022;19(12):1568-1571.

**8.** Jenkinson M, Bannister P, Brady M, Smith S. Improved optimization for the robust and accurate linear registration and motion correction of brain images. *Neuroimage* Oct 2002;17(2):825-841.

**9.** Greve DN, Fischl B. Accurate and robust brain image alignment using boundary-based registration. *Neuroimage* Oct 15 2009;48(1):63-72.

**10.** Satterthwaite TD, Elliott MA, Gerraty RT, et al. An improved framework for confound regression and filtering for control of motion artifact in the preprocessing of resting-state functional connectivity data. *Neuroimage* Jan 1 2013;64:240-256.

**11.** Shafer A, Dazzi F. Meta-analysis of the positive and Negative Syndrome Scale (PANSS) factor structure. *J Psychiatr Res* Aug 2019;115:113-120.

**12.** Crisp CM, Sahni A, Pang SW, Vanes LD, Szentgyorgyi T, Averbeck B, Moran RJ, Shergill SS. Deterioration in cognitive control related mPFC function underlying development of treatment resistance in early psychosis. *Sci Rep* Jun 6 2024;14(1):12985.

**13.** Kitajima K, Tamura S, Sasabayashi D, et al. Decreased cortical gyrification and surface area in the left medial parietal cortex in patients with treatment-resistant and ultratreatment-resistant schizophrenia. *Psychiatry Clin Neurosci* Jan 2023;77(1):2-11.

**14.** Huang H, Jiang Y, Xia M, et al. Increased resting-state global functional connectivity density of default mode network in schizophrenia subjects treated with electroconvulsive therapy. *Schizophr Res* Jul 2018;197:192-199.

**15.** Potvin S, Tikasz A, Lungu O, Dumais A, Stip E, Mendrek A. Emotion processing in treatment-resistant schizophrenia patients treated with clozapine: An fMRI study. *Schizophr Res* Oct 2015;168(1-2):377-380.

**16.** Ahmed M, Cannon DM, Scanlon C, et al. Progressive Brain Atrophy and Cortical Thinning in Schizophrenia after Commencing Clozapine Treatment. *Neuropsychopharmacology* Sep 2015;40(10):2409-2417.

**17.** Alonso-Solis A, Vives-Gilabert Y, Grasa E, et al. Resting-state functional connectivity alterations in the default network of schizophrenia patients with persistent auditory verbal hallucinations. *Schizophr Res* Feb 2015;161(2-3):261-268.

**18.** Kubera KM, Sambataro F, Vasic N, Wolf ND, Frasch K, Hirjak D, Thomann PA, Wolf RC. Source-based morphometry of gray matter volume in patients with schizophrenia who have persistent auditory verbal hallucinations. *Prog Neuropsychopharmacol Biol Psychiatry* Apr 3 2014;50:102-109.
